# *In Vivo* and *in Vitro* Anti-Inflammatory Activity of Neorogioltriol, a New Diterpene Extracted from the Red Algae *Laurencia glandulifera*

**DOI:** 10.3390/md9071293

**Published:** 2011-07-22

**Authors:** Rim Chatter, Rym Ben Othman, Sameh Rabhi, Maria Kladi, Safa Tarhouni, Constantinos Vagias, Vassilios Roussis, Lamia Guizani-Tabbane, Riadh Kharrat

**Affiliations:** 1 Unit of Biotoxines, Pasteur Institut of Tunis, 13, Place Pasteur, B.P. 74. 1002 Tunis-Belvedere, Tunisia; E-Mails: rimchatter@gmail.com (R.C.); satarhouni@yahoo.fr (S.T.); riadh.kharrat@pasteur.rns.tn (R.K.); 2 Laboratory of Immunopathology, Vaccinology and Molecular Genetics (LIVGM), WHO Collaborating Center for Research and Training in Leishmaniasis and International Associated Laboratory (LIA-CNRS), Pasteur Institut of Tunis, 13, Place Pasteur, B.P. 74. 1002 Tunis-Belvedere, Tunisia; E-Mails: rymbenothman@gmail.com (R.B.O.); rabhi_sameh@yahoo.fr (S.R.); 3 Department of Pharmacognosy and Chemistry of Natural Products, School of Pharmacy, University of Athens, Panepistimiopolis Zografou, Athens 15771, Greece; E-Mails: kladi@pharm.uoa.gr (M.K.); vagias@pharm.uoa.gr (C.V.); roussis@pharm.uoa.gr (V.R.)

**Keywords:** anti-inflammatory, neorogioltriol, *Laurencia*, TNFα, NF-κB, NO, COX-2, carrageenan

## Abstract

Neorogioltriol is a tricyclic brominated diterpenoid isolated from the organic extract of the red algae *Laurencia glandulifera*. In the present study, the anti-inflammatory effects of neorogioltriol were evaluated both *in vivo* using carrageenan-induced paw edema and *in vitro* on lipopolysaccharide (LPS)-treated Raw264.7 macrophages. The *in vivo* study demonstrated that the administration of 1 mg/kg of neorogioltriol resulted in the significant reduction of carregeenan-induced rat edema. *In vitro*, our results show that neorogioltriol treatment decreased the luciferase activity in LPS-stimulated Raw264.7 cells, stably transfected with the NF-κB-dependent luciferase reporter. This effect on NF-κB activation is not mediated through MAPK pathways. The inhibition of NF-κB activity correlates with decreased levels of LPS-induced tumor necrosis factor-alpha (TNFα) present in neorogioltriol treated supernatant cell culture. Further analyses indicated that this product also significantly inhibited the release of nitric oxide and the expression of cyclooxygenase-2 (COX-2) in LPS-stimulated Raw264.7 cells. These latter effects could only be observed for neorogioltriol concentrations below 62.5 μM. To our knowledge, this is the first report describing a molecule derived from *Laurencia glandulifera* with anti-inflammatory activity both *in vivo* and *in vitro*. The effect demonstrated *in vitro* may be explained by the inhibition of the LPS-induced NF-κB activation and TNFα production. NO release and COX-2 expression may reinforce this effect.

## 1. Introduction

Natural products derived from plants provide an interesting and promising source for isolating and developing therapeutic molecules to fight against various diseases, including inflammatory ones [1]. In this respect, different natural products derived from marine organisms have been reported to exhibit a broad spectrum of pharmacological activity, including anti-inflammatory effects [2,3].

During the process of inflammation, different cell types are recruited, including monocytes that differentiate locally into macrophages. This leads to the regulated production of various pro- and anti-inflammatory mediators including cytokines, such as TNFα, chemokines and inducible enzymes (COX-2 and iNOS) that play critical roles in controlling the inflammatory processes. The expression of most of these proteins is controlled, at least in part, through the activation of a conserved and ubiquitous transcription factor, NF-κB which plays a key role in inflammatory response.

Several natural products with anti-inflammatory effects have been shown to target and modulate the NF-κB signaling pathway, including a number of terpenoids [4,5]. Molecules belonging to this family were isolated from different marine organisms including coral, sponge and algae [3]. Algae, particularly red algae, represent a rich source of different secondary metabolites, the majority of which consists of acetogenins, halogenated diterpenes and sesquiterpenes [6–8]. Several molecules were derived from the genus *Laurencia* and have been shown to exhibit a number of different activities [9–11]. Using total extracts obtained from different algae, numerous studies have shown associated anti-inflammatory effects both *in vivo* [12,13] and *in vitro* [14,15]. However, to the best of our knowledge, purified molecules with anti-inflammatory effects have not yet been documented from the genus *Laurencia*.

Recently, we have isolated neorogioltriol, a new diterpene with analgesic activity [16] from the organic extract of the red algae *Laurencia glandulifera*. In this report we investigated the anti-inflammatory activity of this molecule both *in vivo*, experimenting on carrageenan-induced inflammation in the rat paw and *in vitro*, based on the expression of NF-κB and COX-2, as well as the release of TNFα and NO, from LPS-stimulated Raw264.7 macrophages.

## 2. Results and Discussion

### 2.1. Effect of Neorogioltriol on Carrageenan-Induced Paw Edema in Rats

The carrageenan-induced paw edema model in rats, one of the well-established acute inflammatory models *in vivo*, was used to test the anti-inflammatory activity of neorogioltriol. In the control group, the intraplantar injection of 100 μL of carrageenan (0.6%) into the rat’s hind paw induced a significant increase in paw edema (Table 1). This observation indicates the development of an inflammatory response and paw swelling (edema). The edema was present as early as one hour after carrageenan injection, progressed rapidly and persisted for at least 5 h after treatment. Both dexamethasone and ASA, used as positives controls, inhibited the oedematogenic response evoked by carrageenan in rats at all assessment times. However, as previously reported [17], the kinetics of the effect of both components was different: whereas the dexamethasone-associated effect was rapid and produced a 42% reduction of the edema after the first hour, the ASA-induced effect was slower (only 17% in the first hour), but reached a 59% inhibition by the fifth hour. Treatment of rats with the neorogioltriol molecule at an intraperitoneal dose of 1 mg/kg significantly reduced paw swelling at all time points tested (Table 1). This reduction was about 28% after the first hour and reached a maximum of 58% at 3 h post injection. Moreover, a 1 mg/kg dose of neorogioltriol proved as effective as 300 mg/kg of ASA in reducing inflammation.

Using the carrageenan-induced paw edema model in rats, our results showed that the red algae-derived natural molecule neorogioltriol was able to reduce the formation of edema in a concentration- and time-dependent manner.

### 2.2. Cell Viability

Cytotoxic potential of neorogioltriol on Raw264.7 cells was tested using the MTT assay. Our result shows that in the presence of up to 250 μM of neorogioltriol, Raw264.7 macrophages viability was not significantly lower than in non-treated cells. We find cytotoxicity only at the highest neorogioltriol concentration (500 μM) which causes an optical density (OD) reduction of about 50%.

### 2.3. Effect of Neorogioltriol on TNFα Release, NO Production and COX-2 Expression in LPS-Stimulated Raw264.7 Cells

Carrageenan-induced rat paw edema has been fully characterized and shown to be associated with the production of several inflammatory mediators [18,19] including TNFα, prostaglandins and nitric oxide release [20–22].

**Figure 2:**
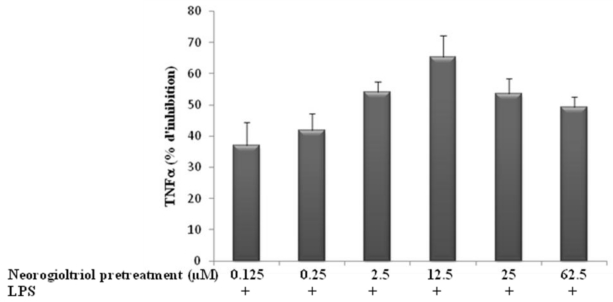
Effects of neorogioltriol on lipopolysaccharide (LPS)-induced tumor necrosis factor-alpha (TNFα) production in Raw264.7 cells. Cells were treated with different concentrations ranging from 0.125 μM to 62.5 μM of neorogioltriol for 30 min prior to LPS (100 ng/mL) stimulation. Supernatants were collected 24 h later and cytokine content was measured by ELISA. Results are expressed as mean values of three independent experiments performed in duplicates, ±standard deviation. Statistically significant differences (*p* < 0.003) between all groups are verified by the Kruskal-Wallis non-parametric test.

We first analyzed the effects of neorogioltriol *in vitro*, on different inflammatory molecules starting with TNFα. The release of TNFα was measured using ELISA assay in the supernatant of LPS-stimulated Raw264.7 cells, pre-treated or not with neorogioltriol for 30 min. The treatment of resting Raw264.7 cells with LPS increased the production of TNFα. For all the concentrations tested, neorogioltriol significantly inhibited TNFα production (Figure 2).

Cyclooxygenase-2 (COX-2) is the key enzyme regulating the production of prostaglandins, the central mediators of inflammation. On the other hand, iNOS activation induces massive NO production at the site of inflammation. We thus investigated the effect of our molecule on LPS-induced COX-2 expression by Western blot and on NO production by measuring the released nitrite in the culture medium by Griess reaction.

**Figure 3:**
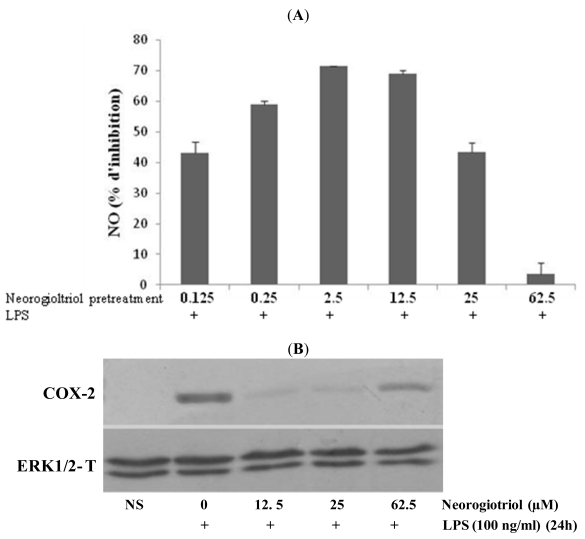
(**A**) The effects of neorogioltriol on LPS-induced nitric oxide (NO) released in Raw264.7 cells. Cells were treated with different concentrations ranging from 0.125 μM to 62.5 μM of neorogioltriol for 30 min and then LPS (100 ng/mL) was added. Supernatants were collected 24 h later and NO content was measured by ELISA. Results were mean values of three independent experiments, performed in duplicates, ±standard deviation. Statistically significant differences (*p* < 0.038) between all groups are verified by the Kruskal-Wallis non-parametric test; (**B**) The effects of neorogioltriol on LPS-induced COX-2 expressions in Raw264.7 cells. Cells were treated with different concentrations (12.5 μM, 25 μM and 62.5 μM) of neorogioltriol for 30 min; then, LPS (100 ng/mL) was added and the cells were incubated for 24 h. Total cellular proteins were resolved by SDS-PAGE, transferred to nitrocellulose membranes, and detected with specific antibodies, as described in the *Experimental Section*. A representative immunoblot of three separate experiments is shown.

Our results show that in murine Raw264.7 cells, LPS treatment (100 ng/mL for 24 h) induces the expression of COX-2 (Figure 3A) and increases NO production 30-fold, as compared to controls. After neorogioltriol pretreatment, the LPS-induced nitrite release was inhibited. At concentrations ranging from 0.125 μM to 12.5 μM, neorogioltriol significantly reduced the level of NO production. However, for neorogioltriol concentrations above 25 μM the curve of NO release turns upward and at 62.5 μM (25 μg/mL) of neorogioltriol, NO release is almost similar to untreated cells (Figure 3A).

The expression of COX-2 was also tested in cells treated with 12.5 μM, 25 μM and 62.5 μM neorogioltriol prior to LPS stimulation. Consistent with the inhibitory effect on NO release, the results (Figure 3B) show that neorogioltriol up to 25 μM inhibited the LPS induced COX-2 expression. However, at 62.5 μM the COX-2 expression is partially restored.

Taken together, our data show that, whereas neorogioltriol has no effect by itself (data not shown), this molecule inhibits the expression of TNFα and COX-2 and the production of NO induced by LPS stimulated Raw264.7 cells. Neorogioltriol however, shows a biphasic effect since this molecule fails to inhibit the expression of key inflammatory mediators, such as COX-2 and NO at the highest concentrations.

### 2.4. Effects of Neorogioltriol on NF-κB Transactivation in LPS-Stimulated Raw264.7 Cells

**Figure 4:**
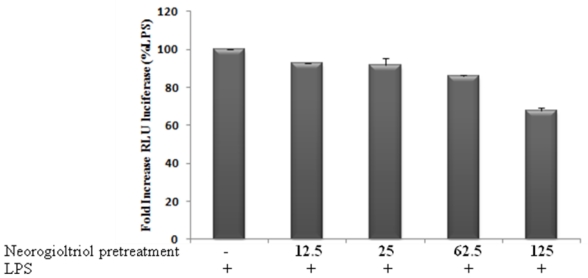
The inhibition of NF-κB activation by neorogioltriol. Cells were stably transfected with a pNF-κB-Luc reporter and then were pretreated for 30 min with different concentrations (12.5 μM, 25 μM and 62.5 μM) of neorogioltriol. LPS (100 ng/mL) was then added and the cells were further incubated for 6 hours. The cells were harvested and luciferase activities were determined in cell lysates and normalized to protein content using a luminometer TD-20/20 (Promega, charbonnieres, France). Results are representative of three different experiments and are expressed as the fold increase in luciferase activity induced by the specific experimental condition, relative to the luciferase activity measured in LPS stimulated cells. Statistically significant differences (*p* < 0.01) between all groups are verified by the Kruskal-Wallis non-parametric test.

The anti-inflammatory potential of terpenes has been linked to their capacity to suppress NF-κB signaling [4,23] which coordinates the expression of proinflammatory enzymes and cytokines including iNOS, COX-2 and TNFα [24–26]. Neorogioltriol was thus evaluated *in vitro* for its ability to inhibit LPS-mediated NF-κB transcriptional activity. Raw264.7 cells, stably transfected with pNF-κB-luc plasmid, containing three NF-κB target sequences linked upstream to the luciferase reporter gene, were either stimulated with LPS or treated with different concentrations of neorogioltriol prior to LPS stimulation. Our result shows that LPS induces NF-κB activation. The pre-treatment with neorogioltriol prior to LPS stimulation significantly decreased LPS induced NF-κB transactivation (Figure 4). This result shows that the anti-oedematogenic effect of neorogioltriol correlates with the suppression of NF-κB activation.

However, despite reducing NF-κB activity, high concentrations of neorogioltriol fail to inhibit the expression of certain NF-κB-dependent genes that are relevant to the inflammatory process, such as COX-2. These results suggest that the observed loss of anti-inflammatory efficacy at high doses of neorogioltriol was independent of NF-κB or indirectly dependent on NF-κB inhibition.

### 2.5. Effect of Neorogioltriol on MAPK in LPS-Stimulated Raw264.7 Cells

The mitogen-activated protein (MAP) kinases play a key role in the regulation of cellular response to cytokines and stresses and are also known to be important for NF-κB activation. We thus tested whether the observed loss of anti-inflammatory efficacy may be mediated through MAPK activation.

**Figure 5:**
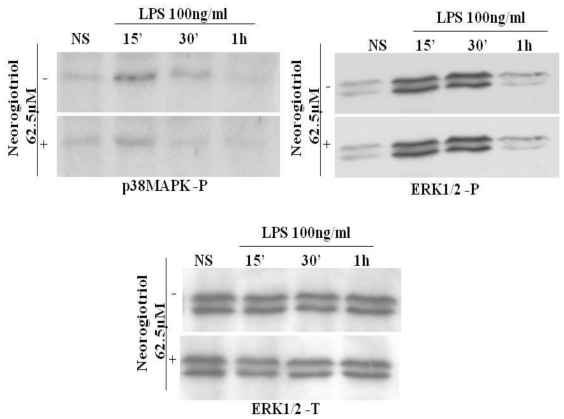
Effect of neorogioltriol on MAPK activation in LPS-stimulated Raw264.7 cells. Cells were pretreated for 30 min with 62.5 μM (or 25 μM) of neorogioltriol. LPS (100 ng/mL) was then added and the cells were further incubated for indicated times. Cells were collected and subjected to Western blotting with antibodies specific for phosphorylated forms of p38 (p38MAPK-P) or ERK1/2 (ERK1/2-P). Total ERK immunoblots were shown as loading control; similar results were obtained for the two concentrations of neorogioltriol used. Results are representative of two independent experiments.

We first studied the effect of neorogioltriol on LPS-induced MAPK activation. Our results show that at the highest concentrations used (25 μM and 62.5 μM), the neorogioltriol molecule fails to interfere with LPS-dependent ERK activation and only slightly inhibited the p38 MAPK phosphorylation (Figure 5). Moreover, the inhibition of MAPK pathways by PD98059 or SB203580 treatment did not alter the capacity of neorogioltriol to inhibit the LPS-induced NF-κB transactivation (data not shown).

MAPKs have been reported to be involved in the LPS-induced iNOS expression signaling pathway [27] which regulates the production of NO which, in turn, may enhance the expression of COX-2. On the other hand, at the highest concentrations used, neorogioltriol does not display a significant inhibitory effect on MAPK phosphorylation. Using SB203580 (or PD98059), we thus tried to see whether or not MAPK activity may explain the recovery of NO release by the cells treated with the highest concentrations of neorogioltriol.

**Figure 6:**
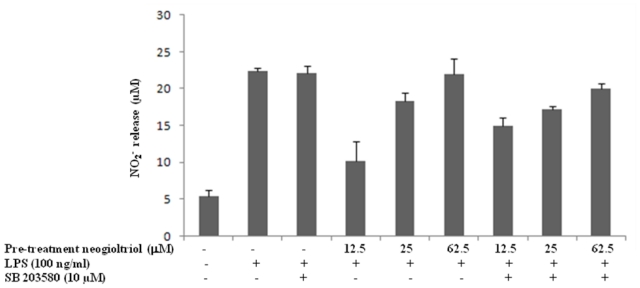
Effect of p38 MAPK inhibitor on NO release in neorogioltriol treated Raw264.7 cells. Cells were pretreated for one hour with SB203580 (10 μM) and then with neorogioltriol (12.5 μM, 25 μM or 62.5 μM) before LPS stimulation. Supernatants were collected 24 h later and NO content measured by Griess reagent. Results were mean values of two independent experiments, performed in duplicate, ±standard deviation. Comparisons between SB203580 treated and non treated cells were made using the non-parametric Mann-Whitney test, with significance of *p* < 0.05.

Raw264.7 cells were incubated for one hour with SB203580 (or PD98059) prior to neorogioltriol treatment and LPS stimulation. Our results show that the use of p38MAPK (or ERK1/2) inhibitor does not inhibit the recovery of NO production observed with the highest concentration of neorogioltriol (Figure 6) suggesting that abrogation of NO inhibition in the neorogioltriol treated cells is not dependent on MAPK activation.

Taken together, these results show that the effect of neorogioltriol at high concentration is independent of MAPK and NF-κB. This effect may however be indirectly dependent on NF-κB inhibition. Indeed, some non steroid anti-inflammatory drugs (NSAIDs) are known to activate COX-2 through signaling pathways independent of NF-κB and MAPK and involving the nuclear factor PPARγ. On the other hand, LPS has been shown to drive down PPARγ expression through the activation of NF-κB [28]. This may suggest that the repression of NF-κB by neorogioltriol inhibits the negative loop of NF-κB on PPARγ, which may allow the latter to induce the expression of NOS2 and COX-2. We are currently investigating this hypothesis.

## 3. Experimental Section

### 3.1. Isolation of Neorogioltriol

The collection of *Laurencia glandulifera* and the extraction and isolation procedure of neorogioltriol have been already described [16].

### 3.2. Carrageenan-Induced Paw Edema

The anti inflammatory activity on carrageenan-induced paw edema was determined according to the method described by [29]. Naïve rats were randomly allocated to four groups and pretreated 1 hour before intraplantar administration of 100 μL of 0.6% of carrageenan. (i) Control group: received 2.5 mL/kg of vehicle (physiological solution (0.9% NaCl)) used to re-suspend the different drugs (ii) Standard group 1: received 1 mg/kg of dexamethasone (2.5 mL/kg); (iii) Standard group 2: received 300 mg/kg of asprin (2.5 mL/kg); and (iiii) Test group: received 1 mg/kg or 0.5 mg/kg of neorogioltriol (2.5 mL/kg). The drugs were administrated by intraperitoneal injection. The edema volume was determined using a plethysmometer (model 7150, Ugo Basile, Italy) prior to and 1, 3 and 5 h after carrageenan injection. The percentage of inhibition in treated rats *versus* control was calculated using the following formula: EI (%) = ([(*C*_t_ − *C*_0_) control − (*C*_t_ − *C*_0_) treated]/(*C*_t_ − *C*_0_) control) × 100 where *C*_t_ correspond to edema volume after carrageenan injection and *C*_0_ correspond to edema volume before carrageenan injection.

### 3.3. Reagents and Antibodies

ERK1/2 antibodies and p38 MAPK antibodies were from Ozyme (Ozyme, Saint Quentin en Yveline, France). COX-2 antibody was from Becton Dickinson (BD Biosciences, France). PD098059, SB203580, LPS and griess reagent were purchased from Sigma (Sigma-Aldrich, Taufkirchen, Germany).

### 3.4. Cells Culture

The mouse macrophagic cell line Raw264.7 cells were obtained from American Type Culture Collection and were grown at 37 °C, in RPMI 1640, supplemented with 5 mM L-glutamine, 10% heat inactivated fetal calf serum (endotoxin-free; HyClone), penicillin (100 U/mL) and streptomycin (100 μg/mL).

### 3.5. Cytotoxicity Test

The MTT assay was used to test the potential toxic effects of neorogioltriol on the mouse macrophagic cell line Raw264.7 cells. Briefly, Raw264.7 cells were incubated with various concentrations of neorogioltriol for 24 h. MTT solution was added to cell cultures at the final concentration of 0.5 mg/mL and cells were incubated for an additional 2 h. Thereafter, medium was removed and plates were thoroughly washed with PBS. Cells were finally lysed in DMSO. The absorbance of each well at 540 nm was read by an automatic microplate reader. The average OD measured at different concentrations of neorogioltriol was compared using one-way analysis of variance (ANOVA). Proliferation of the cells exposed to the drugs was compared to the negative control. *P* < 0.05 was considered statistically significant.

### 3.6. Transfection and Luciferase Assay

Raw264.7 cells were stably transfected by electroporation with an NF-κB luciferase reporter construct containing three NF-κB target sequences in tandem fused to the luciferase reporter gene (3X-NF-κB-Luc), a gift from Dr J. Pierre (Faculté de pharmacy, Chatenay-Malabry, France). Cells were either left untreated or were treated with neorogioltriol at different doses for 30 min, they were then washed and stimulated with LPS (100 ng/mL) for an additional 6 hours. Protein concentration was determined by microBCA assay. Luciferase activity was determined using a luminometer TD-20/20 (Promega, charbonnieres, France).

### 3.7. Western Blot Analysis

Cell extracts were obtained by adding 25 μL of lysis buffer containing 10 mM Tris-HCL, pH 7.5, 50 mM NaCl, 50 mM sodium fluoride (NaF), 2 mM EDTA, 1 mM ethylene glycol-bis (β-aminoethylether)-*N*,*N*,*N*′,*N*′-tetraacetic acid (EGTA), 2% Nonidet-P40 (NP-40), 0.75% sodium deoxycholate (DOC), 1 mM orthovanadate, 1 μg/mL aprotinine, 1 mM PMSF, 1 mM DTT. After 30 min incubation on ice, the extracts were centrifuged at 15,000 rpm for 20 min. 25 μg of whole cell lysates were resolved by electrophoresis in a 12% SDS-polyacrylamide gel. Resolved proteins were electrophoretically transferred onto PVDF sheets (Hybond-P; Amersham) and membranes were blocked by incubation in 3% non-fat milk and 0.1% Tween in PBS for 1 h at room temperature followed by incubation with COX-2 antibody in 0.1% Tween-PBS. Bound antibody was detected by incubation with horseradish peroxidase-coupled secondary antibody (Amersham Pharmacia Biotech., Buckinghamshire, UK). To ensure equal loading, the blots were then stripped (62.5 mM Tris, pH 6.8), 0.1 M β-mercaptoethanol, 2% SDS) and re-probed with ERK1/2 antibody.

### 3.8. Measurements of Nitrite Production and Cytokine Assays

Macrophage cells were seeded in 24 wells, treated with different concentrations of neorogioltriol or vehicle for 30 min and then incubated with 100 ng/mL of LPS for 24 h. NO production in culture supernatant was spectrophotometrically evaluated by measuring nitrite, an oxidative product of NO. Nitrites were measured by Griess reaction by mixing 50 μL of culture supernatant with 50 μL of griess reagent. Absorbance was measured in microplates at 540 nm against a calibration curve with sodium nitrite standard. Supernatants were also analysed for TNFα content using BD Pharmingen (BD Biosciences, France) antibodies. The assays were performed according to the manufacturer’s instructions.

### 3.9. Statistical Analysis

For TNFα production, NO release and NF-κB activation, the Kruskal-Wallis test was used to analyze significant differences between groups. The comparison between the two groups (presence or absence of SB203580) was performed using the Mann-Whitney test. For all statistical comparisons a *p* value <0.05 was defined as significant.

## 4. Conclusions

As far as could be ascertained, this is the first report that shows anti-inflammatory activity for a purified algal metabolite. We have demonstrated that, in addition to its analgesic activity [16], neorogioltriol exhibits anti-inflammatory activity *in vivo*. This effect may be explained by the capacity of neorogioltriol, at all concentrations tested, to inhibit NF-κB transactivation and TNFα release. The inhibitory effect of this compound on COX-2 expression and NO release is likely to reinforce the anti-inflammatory effect of neorogioltriol at concentrations below 62.5 μM. However, the exact mechanism of neorogioltriol action needs to be clarified and the target molecule in the cell identified.

## Figures and Tables

**Figure 1:**
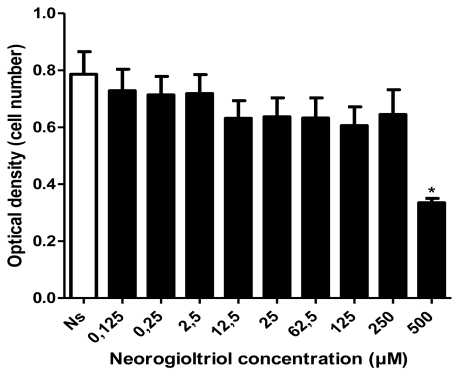
Raw264.7 macrophages were either left untreated or were treated with various concentrations of neorogioltriol. The cytotoxic effects were recorded at an observation period of 24 h by using 3-(4,5-dimethylthiazol-2-yl)-2,5-diphenyl tetrazolium bromide (MTT) test as described under “Experimental Section”. The cell number in each well was recorded in the form of optical density (OD). Data are expressed as means ± SD of three independent experiments performed in triplicates. Results were analyzed by using one-way analysis of variance (ANOVA) with significance of *p* < 0.05.

**Table 1:** Effect of neorogioltriol on carrageenan-induced paw edema in rats.

Times after carageenan injection (h)				1 h		3 h		5 h	
Groups	Treatment	*n*	Dose (mg/kg)	EV	EI	EV	EI	EV	EI
				(10^−2^ mL)	(%)	(10^−2^ mL)	(%)	(10^−2^ mL)	(%)
Control	Control	6	-	18.3 ± 2.7	-	56.3 ± 5.8	-	69 ± 7.7	-
Tests	Neorogioltriol	6	0.5	15.5 ± 3.1	15.3	42 ± 5.5 (*)	33.3	49.1 ± 6.1	28.8
			1	14.1 ± 3 (*)	22.95	23.5 ± 3.7 (***)	58	33 ± 3.5 (**)	52.27
References	Aspirin (ASA)	6	300	16.5 ± 3.1	9.83	25.1 ± 6.7 (**)	55.17	28.1 ± 2.2 (**)	59.27
	Dexamethasone	6	1	9.83 ± 1.8	47.54	39.3 ± 3.8 (***)	42.5	32.5 ± 5.7 (***)	52.86

Neorogioltriol (0.5 or 1 mg/kg) was administrated one hour before the intraplantar carrageenan injection. Reference animals were treated with dexamethasone (1 mg/kg) or aspirin (ASA) (300 mg/kg); control animals received vehicle (0.9% NaCl) used to re-suspend the different drugs. Six animals were used for each treatment group. The drugs were administrated by intraperitoneal injection. The edema volume (EV) was determined immediately before and 1, 3, and 5 h after the carrageenan injection. When needed, the edema inhibition (EI) was calculated. The percentage of inhibition in treated rats *versus* control was calculated using the following formula: EI (%) = ([(*C*_t_ − *C*_0_) control − (*C*_t_ − *C*_0_) treated]/(*C*_t_ − *C*_0_) control) × 100 where *C*_t_ correspond to edema volume after carrageenan injection and *C*_0_ correspond to edema volume before carrageenan injection. The values are expressed as the mean ± SD;

* *p* < 0.05;

** *p* < 0.01;

*** *p* < 0.001 determined by Student’s *t* test compared with control.
